# The Potentials of Digital Workplace Health Promotion

**DOI:** 10.3390/ijerph21070902

**Published:** 2024-07-10

**Authors:** Elisabeth Nöhammer, Michaela Drexel

**Affiliations:** 1 Department of Public Health, Health Services Research and HTA, UMIT TIROL—Private University for Health Sciences and Health Technology, 6060 Hall in Tirol, Austria; 2 Krankenhaus der Elisabethinen GmbH, Elisabethinergasse 14, 8020 Graz, Austria; michaela.drexel@elisabethinen.at

**Keywords:** Digital Workplace Health Promotion, employees, barriers, benefits, impact, hospital, healthcare

## Abstract

Workplace Health Promotion (WHP) can sustainably impact organizations by improving employee health and strengthening legitimization. Digital Workplace Health Promotion (DWHP) may have even more impact thanks to its scope. This study reports on a hospital in Austria wherein DWPH was introduced into the existing WHP structure in combination with a digitalization effort for the entire organization. The approach was mainly quantitative with a few open questions and included a survey before and an evaluation after the project with about 240 respondents each. The use, intentions, barriers and benefits of DWHP from the employees’ perspectives were reported on to evaluate the potentials of DWHP for furthering sustainable developments within organizations. While DHWP is perceived as positive, current use is low. Nevertheless, intended future use is promising and perceived benefits are higher after implementation. However, perceived barriers are still high, requiring organizational efforts.

## 1. Introduction

Digitalization has received tremendous interest in practice and research in recent years, leading to various conceptualizations [1,2]. In this study, we define it as “the transformation of the socioeconomic environment through processes of digital artifact adoption, application and utilization [2] (p. 17)”. This not only includes the transformation of analogous data into digital ones [1], but also the intensified use of Information and Communication Technology (ICT) and the restructuring of life domains by the latter [3,4]. Following Steinmueller’s work on the codification of knowledge [5], Day et al. [6] define ICT as “any electronic device or technology that has the ability to gather, store, or send information” (p. 473). Due to the high availability and use of devices like mobile phones, smartphones and laptops, and digital technologies like the Internet, emails and artificial intelligence [7,8], ICT is paramount in society.

Increased use of ICT comes with highly interdependent opportunities and risks for individuals and organizations [9,10,11,12]. The (im)balances of ICT chances and challenges at the workplace become very apparent in employee health. This is because organizations influence population health via the working conditions for their employees, the quality and safety of their products and services as well as their socio-ecological effects [13]. Recent research suggests to combine investigations on ICT, health at the workplace and digital efforts to promote the latter [14].

While employee health protection is crucial in Organizational Health and Safety Management (OHS, OHSM) in general, health and wellbeing at the workplace comprises much more than complying with health, hygiene and safety regulations. Workplace Health Promotion (WHP) means “the combined efforts of employers, employees and society to improve health and well-being of people at work” (p. 2) by adapting the work setting with its structures and processes to be more health-promoting, increasing employee participation and strengthening individual development [15]. So far, however, WHP in a comprehensive sense is not very common [16,17], with small and medium-sized organizations being especially challenged [18]. Initiatives range from comprehensive employee health programs to single activities in specific areas [19]. Both can include interventions and offers, e.g., regarding nutrition, risk behaviors like smoking, physical activity [20,21], but also mental health [22] and improvement in the work setting regarding its structures, processes and organizational culture [23].

Digitalization can be of great aid in establishing and offering WHP within the organization [24]. Especially Digital Workplace Health Promotion (DWHP), the “use of digital means and tools to create and deliver health interventions for employees” [14] is very promising, due, among others, to its better reachability especially of health-wise easily underserved populations like shift workers [25], thereby reducing health-related inequalities and working toward reaching the UN’s Sustainable Development Goals [26,27,28]. In addition, the personalization of employee health offers is often easier [29], cost-efficient and spill-over effects toward society are expected [14]. Also, the applicability of DWHP is broad and can cover all aspects of health [30,31], e.g., physical activity [32], medical checks [33], mental health [34] and eating habits [35]. Offers include apps [33,34,36,37,38], wearables, fitness trackers [39], websites, educational videos, social media, messages, phone calls and a combinations of the above [31].

While many aspects of digitalization and sustainability have already been studied [40], the impact of digital employee health offers is a new research area and its contribution to transforming an organization into a healthier one still warrants investigation [14,30,35,37,41,42]. Moreover, investigations regarding the benefits to the workforce are needed [14,43]. Said workforce should perceive DWHP positively and it should match its requirements [14]. This is also required as most data on employee preferences regarding WHP were collected before the pandemic, making it unclear which topics they would like to see covered [44], be it in a traditional on-site or digital manner.

In this study, the focus is twofold: (1) we investigate the perspective of the workforce regarding DWHP and (2) the potentials of DWHP for transforming the organization into a healthier one. For this to be the case, DWHP needs to be used and rated favorably, which requires a positive ratio of benefits over barriers (see Hypotheses in Section 2 and Section 3.2).

An overview of workplace health initiatives, their reasoning and barriers, is given in the following section to contextualize DWHP and its potentials. After this, the setting of the study and the methods employed are described. Section 4 is structured thematically to not only focus on the established hypotheses but also cover the topic of DWHP as perceived by the employees. Together with the discussion of the results, this study outlines future research requirements based on the findings as well as its limitations. Section 6 contextualizes this study and highlights the necessity of discussing the role of organizations for employee and public health.

## 2. Background and Hypotheses

Health is assumed to be a basic value [45] and access to healthcare is considered a social right [46]. While human health can be seen as a fundamental need on a societal and individual level, organizations address this differently, using the full continuum from immersion to disregard. Via laws and standards, a certain degree of health protection is mandatory in most states [47]. As Oeij and colleagues put it: “(s)ocial and human values regarding health and wellbeing are seen as conditional to human rights and decent work” [48] (p. 284) which should also apply to digitalization in the work context [48,49]. When organizations are viewed as agents for producing goods and services for societal needs, demanding health protection and ideally also health creation at work is a rational consequence. The organization’s existence would be endangered should its actions be counterproductive for the need tackled. Moreover, empirical evidence shows the impact of employee wellbeing on positive outcomes for the organization, thus promoting organizational sustainability [50]. Based on these considerations and the societal importance of companies, becoming a health-promoting organization would be sustainable inwardly and outwardly [51].

Organizational health development is creating and improving health in structures (capacities on the individual and organizational level) and their interactions (processes) in a targeted as well as continuing general way [13,52]. With the objective being the creation of a healthy, sustainable organization, which operates successfully on the market but also internally regarding employee wellbeing [53], there are at least four levels to consider for workplace health interventions: the individual, the group, the organization and processes within the latter [54]. This is often achieved via initiatives of WHP. Implementing these successfully depends on management processes being in place [55], but also on value congruence with health promotion such as internal capacity criteria and evidence for the WHP program elements [56]. In addition, they need to be aligned with the needs and values of the employees [23,57,58]. A comprehensive program is suggested that includes offers concerning the salutogenic improvement of the setting and health-related behaviors of the target group [23]. Thus, this can also mean combining offers for all employees with specialized ones [59]. It is important to note here that participation rates can be calculated for behavior-oriented offers, but hardly for setting-oriented offers, as the latter are designed to change the environment for all. This could be healthy food at the canteen or digital ways to prevent mental overload, e.g., by blocking emails during certain times of the day. This means that a health program at the workplace, or at least aspects of it, can reach all employees and highlights the importance of salutogenic processes and structures in organizations. For this, organizational awareness of health impacts for employees and society must be granted.

A salutogenic orientation, and even health promotion at work, are voluntary for both employer and employees. Due to this, programs are not always ideal [17], participation rates are often low [60,61,62] or attract mainly those who are already trying to lead a healthy lifestyle [63,64]. Fully reaching the potentials of WHP for employees and organizations, however, would require optimizing the participation rate [65,66,67,68,69,70,71,72,73]. Expert advice on this [56,74,75] and models [76] are available and require adaptation to the individual organization. Though general implementation science can be drawn upon [77,78,79], aligning a program to specific internal needs and target groups can be laborious. Therefore, having employees participate in setting WHP is often suggested [57,80], for example, via health circles [81], as this can be more efficient.

DWHP can alleviate the issues above for both the employer (see cost-saving options described in the Section 5) and employees, who are the focus of this study. Digital offers can increase the range of participants by being available off-site or attracting those who do not want to participate in person [82]. Also, those who are hindered time-wise or by low social support at the workplace [83] can be reached. These considerations lead to the expectation that staff rates the availability of DWHP offers positively, defining H1 as follows:

**H1.**  *DWHP is rated favorably by the employees.*

This study was conducted in a hospital (the details of which can be found in the next section), as these are among the organizations that could benefit the most from DWHP. Their staff is very interested in health [57], highly burdened [84] and has irregular working hours. Shifts are common and part-time work is high, plans must be adapted rapidly in case of emergencies, making attending WHP offers very challenging for many employees [85]. This holds true for regular offers, but also one-time events. As hospital staff is thus acutely aware of the barriers towards using WHP, we posit the following:

**H1a.**  *DWHP use is high.*

Moreover, we assume that, with the current global personnel shortages in healthcare [86] and resulting time pressures and psychological burden [87], intended future participation in DWHP will be higher than current use. As intention of use has been shown to relate to later participation [88], we also intend to test the following:

**H1b.**  *Intended use of DWHP is high.*

As has already been reported for digital applications in the context of WHP, the limitations of regular offers like time barriers are less prevalent while positive attributes like personalization can be enhanced [36]. Moreover, research in Japan found that employees with experience using digital health services (here: mental) were more likely to access these via their employer rather than privately [34]. Therefore, we assume that experience with DWHP has an effect and that

**H2.**  *The perceived benefits of DWHP increase over time.*

**H3.**  *The perceived barriers for DWHP use decrease over time.*

## 3. Materials and Methods

As determinants of health are multifaceted [89], efforts to promote health need to be specified for the respective setting. For this study, we report on a hospital in Austria (~500 employees at the start of the study, currently: 660) with an established WHP program [57] which was supplemented with DWHP. This was achieved as WHP use data show inequalities in opportunities to profit from the offer, mainly due to the very divergent working times of the staff. The organization was chosen based on three considerations: (a) employees needed to be familiar with WHP, and DWHP needed to be a newly introduced, (b) the organization had to be interested in DWHP, and (c) as WHP and DWHP are not mandatory in Austria, there needed to be funding for WHP and DWHP. In addition and to comply with the settings approach of health promotion [90], the target group for both WHP and DWHP had to be all employees.

However, being a hierarchical organization, not all staff is extensively trained to use digital appliances or has access to them. Therefore, training needs must be assessed and DWHP offers should be as easy to use as possible and match what people know from other life areas, e.g., video clips as downloads from the intranet. Moreover, digitalization efforts in HR and marketing led to considerations regarding the digital competences of staff. It became clear that digital competences may be unequally distributed and thus the impact of DWHP may be endangered, especially in the case of structural and competence inequalities coinciding.

The sustainable transformation of organizations requires the employees to be integrated as vital stakeholders, especially when digitalization is concerned [91]. Including the target group in the design, implementation and evaluation of a health-promotion program also long been used and is highly advised in order to increase fit and acceptance [92] and to ensure that the offer fits to the target population and the objectives can be reached [23]. Participation is highlighted in implementation guidelines which are typically based on behavioral change models [93,94,95] and/or closely linked to change management principles [96,97,98] or best practice guidelines [56,99,100]. In a stepwise manner, doing a needs assessment, developing the intervention plan and objectives, implementing the intervention and evaluating it are required [92].

Therefore, we followed this approach in our research process: (1) Before the start of DWPH and to cover the exact needs in the context of digitalization, all employees were asked to fill in a questionnaire in May 2022 regarding their perceptions of the digitalization climate within the organization, the extent to which they felt exposed and ready to work with digital equipment, plus wishes and barriers regarding DWPH (see details in Section 3.1. and Appendix A). As they were familiar with WHP, they could be surveyed regarding which offers they wanted in a digital way. In the next step, (2) DWHP elements were designed and introduced. These included behavior-oriented parts like exercise videos and hybrid trainings on mindfulness, as well as an online nutrition workshop, which were all recorded and uploaded to the intranet (employee portal). Moreover, there were setting-oriented parts as, e.g., hardware adaptations, implementing a new digital communication platform (employee portal) and enlarging this with e-learning offers.

The regular WHP program was also continued, including setting-oriented changes like redesigning a room for relaxing, and behavior-oriented interventions like swimming, health days, eye health checks, back exercises, trainings in de-escalation and leadership, on-site workshops and trips. Covering both setting-related as well as behavior-related aspects is essential in health promotion to ensure a sustainable impact.

After the implementation phase, the offer was (3) evaluated (see details in Section 3.2 and Appendix B). This was achieved by integrating the evaluation of DWHP into the regular evaluation of WHP and the psychological burden at the workplace in November 2023, creating a rich database.

### 3.1. First Survey: Wishes, Needs and Attitudes

DWHP is a new topic; therefore, a questionnaire (see Appendix A) was designed based on the literature and prior research [14]. Items on specific plans and digitalization endeavors of the organization (e.g., an employee portal) were added based on collaborative work of the first author as external scientist and advisor and the second author as internal project lead. This approach was chosen as there is no standardized tool available [30], not even for WHP [101]. This is because WHP programs need to be targeted to the issues of the employees and thus have different objectives [102,103,104,105]. This makes this study a first suggestion also for other organizations engaging in DWHP as the content can be adapted. To ensure maximum usability, the survey was provided as paper and pencil plus as an online version. All employees were invited to fill in the survey. Participation was completely anonymously and was non-mandatory. The surveys were all completed in German in May 2022. The paper and pencil versions were collected in boxes in the organization and then sent to the university of the first author where they were combined with the online questionnaire data to form a final dataset for analysis. This was done to ensure that the employees knew their data were protected and to accommodate their need for privacy and anonymity and reduce the likelihood of biases like social desirability.

The survey comprised three parts (see Appendix A):

1. General questions about the digital representations of the organization inwardly and outwardly (website, employee portal) and their perception by the employees; the amount of digitalization in the daily work of the employees plus ratings of burden vs. chances; digital competences and wishes for training.
2. DWHP: Rating of current (i.e., online booking of offers) and future offers (i.e., digital offers), comparison of WHP and DWHP; determinants and barriers of using DWHP; wishes regarding DWHP. Ideas for potentially interesting digital offers were derived from a prior project at the hospital [57].
3. Demographics (age group, occupational group, working times, education).

Most questions were quantitative, those with thematic background (parts 1 and 2) were 5-point Likert-scaled with a neutral option. Items formulated as statements were scaled regarding agreement, questions concerning WHP/DWHP differentiated regarding attractiveness.

Examples:

- I have so many digital parts in my daily work that I do not want digital WHP offers. (agree/rather agree/neither-nor/rather disagree/disagree)
- How attractive would digital LIVE-offers of WHP be for you in the following areas? (e.g., dietary habits?) (very attractive/rather attractive/neither-nor/rather not attractive, not attractive)

Open questions were specific wishes, suggestions or possibilities to further explain something, and available in Parts 1 and 2.

Upon completion of this survey and internal discussion, the hospital continued and adapted its WHP program plus started providing digital offers, mainly videos and hybrid workshops plus hardware adaptations and enlarging the offer available in the employee portal (more details seen above under Section 3).

### 3.2. Second Survey: Evaluation

Contrary to the first survey which was conducted as stand-alone investigation, the evaluation (see Appendix B) was integrated—as the first part of the questionnaire—into the evaluation of psycho-social burden at the workplace in 2023. This was only offered in the paper and pencil version which again was collected in boxes in the organization and then sent to the university of the first author. This approach had to be taken to avoid biases like social desirability and to prevent a low response rate in case of two separate surveys. As positive side-effect, the dataset allows for more detailed analyses especially since it traditionally also integrates detailed evaluations of WHP, e.g., general satisfaction levels, impact and areas of interest [57,82,106]. However, the scaling had to be adapted to a 4-point Likert-scale to fit to the bigger survey for the questions that were repeated from survey 1. Also, the scaling for age was less detailed compared to the first survey to confirm the usual survey on psycho-social burden at the workplace. Questions on frequency were scaled as very often–often–occasionally–seldom–never.

Newly added questions for the project refer to appraisal focused on the comparison of satisfaction levels with WHP vs. DWHP to allow for more detail in the next (D)WHP program cycle. Items that were kept from survey 1 (see items in italics in Appendix B) refer to difficulties in finding digital information, use and evaluation of this information in the context of DWHP, current and planned use of (D)WHP, benefits and limitations of DWHP and digital information options. The evaluation questionnaire (see Appendix B) consists of five parts covering WHP and DWHP, their use, interest in specific offers, a general rating and demographics.

Based on the intentions and design of this study, we aim to answer the research question of whether DWHP does have the potential to contribute to the sustainable transformation of organizations. This is based on employee acceptance and use. Therefore, we will analyze the proxies for this and posit the hypotheses described above.

## 4. Results

The presented results we focus on are those of the second survey. This is because the research questions are mainly found here, and more demographics were asked as the intention of the first survey was to gain an overview of the DWHP-related needs. However, data from the first survey are included in square brackets for identical questions and mentioned explicitly in the text. Please note that values shown in figures are rounded. 

### 4.1. Demographics

When the project application was submitted, the hospital had 529 employees. The first survey received 241 responses, which regarding the baseline equals 45.56%. At the time of the project’s end, the hospital counted 660 employees. The second survey, the evaluation, was filled in by 254 employees, which based on 660 employees is a response rate of 38.5%. The demographics are listed in Table 1.

In the following sections, responses indicating agreement (e.g., agree, rather agree/applies, rather applies) are combined. In case a different scaling was used, this is mentioned in the text. The major demographic elements are tested regarding their influence using Chi2-Tests as most demographic questions were nominally scaled. The Chi2-Tests gives “information not only on the significance of any observed differences, but also provides detailed information on exactly which categories account for any differences found” [107] (p. 143).

### 4.2. Importance and Knowledge about DWHP

To contextualize the findings, respondents were asked to rate the importance WHP for them and their general perception of the on-site and digital offer. WHP was rated as generally important topic by 95.2% of the respondents. Moreover, 85% stated that it was also personally important. Over 80% (82.6%) [2022: 71.3%] knew where to find information on the WHP offer and over 65% (65.1%) [2022: 50.7%] used the employee portal (intranet). According to the Chi2 test, this also tends to apply primarily to employees over the age of 45 (p = 0.002). Digital information options on occupational health promotion were described as easy to use (general: 73.6% [2022: 56.4%]; digital registration: 77.1% [2022: 56.4%]). This was reported mainly by respondents without patient contact (Chi2 test with *p* = 0.025).

More than half of respondents knew which DWHP services were available in the company (54.8%). Also, 83.9% stated that they knew where to find information on WHP in the employee portal. However, the latter applies more to people who had been with the company for more than 3 years or were older than 45 (Chi2 test, each at *p* = 0.000). About 75% stated that DWHP is accessible for everyone, though it becomes apparent that complete agreement stems mainly from participants over 45 years of age (*p* = 0.008).

A total of 74.1% stated that the information on WHP offers reached every person in the company. For the latter question, the Chi2 test shows a significant indication that full agreement comes primarily from people over the age of 45 (*p* = 0.008).

General improvements in working conditions due to WHP in the company were felt by 45% of respondents. According to the Chi2 test, people with no patient contact (*p* = 0.000) are more likely to say that they do feel improvements due to WHP in the company.

### 4.3. Personal Rating of (D)WHP

**Figure 1:**
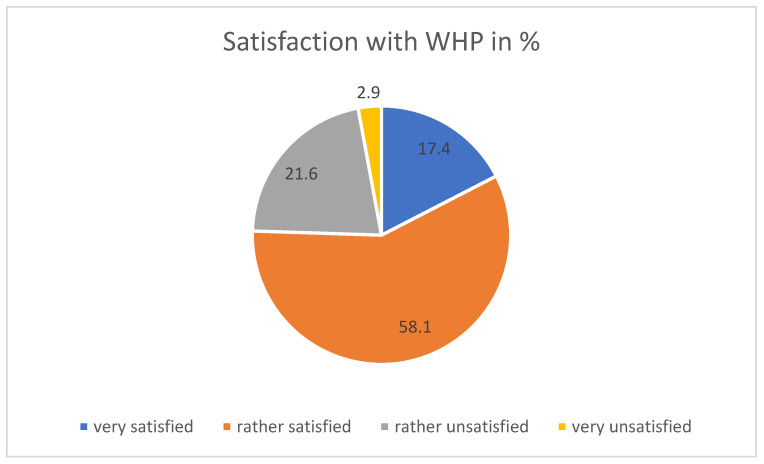
Satisfaction with WHP offer (overall value).

Overall, three-quarters (75.5%) of people were very or rather satisfied with the WHP offer (see Figure 1).

People over the age of 45 were significantly more likely to be very satisfied (Chi2 test). In the open responses (see below), it was mentioned that an adaptation of the services to younger people and thus higher fitness levels could be beneficial. Just under 22% (21.6%) were rather dissatisfied. Only 2.9% stated that they were not satisfied, with a slight tendency toward shift workers (Chi2 test, *p* = 0.017).

**Figure 2:**
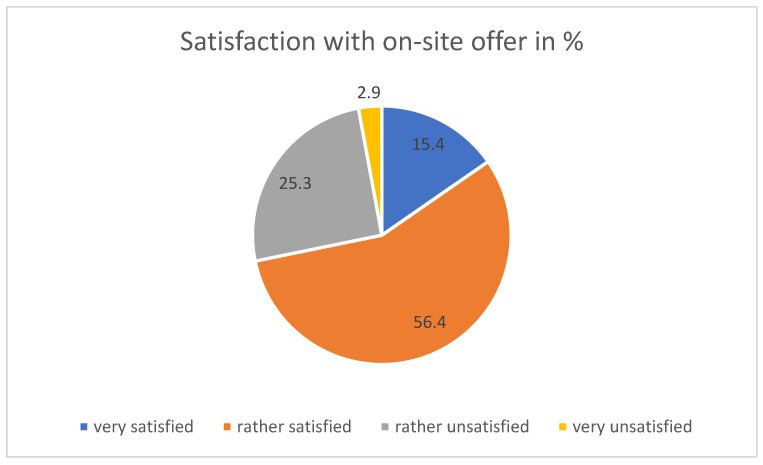
Satisfaction with on-site WHP offer.

Regarding the on-site services (i.e., WHP; see Figure 2), 71.8% were very or rather satisfied, especially if there had no patient contact or if they were over 45 years of age (Chi2 test).

**Figure 3:**
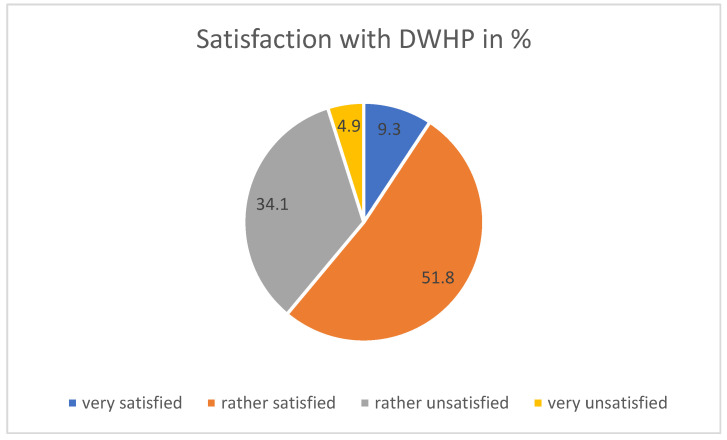
Satisfaction with digital WHP offer.

At 61.1%, satisfaction with DWHP is around 10 percentage points lower, but still very positive (see Figure 3). Moreover, 73.3% [54.8%] saw DWHP as a good addition to WHP, and according to the Chi2 test this tends to be higher among people with on-call duty (*p* = 0.049).

Thus, H1—DWHP is rated favorably—is confirmed. Here too, satisfaction tends to be higher among people without patient contact (*p* = 0.014).

However, 56.8% [26.6%] of respondents were convinced that WHP should only take place live. A total of 48.1% [29.9%] stated that there were so many digital offers in their daily work that they did not want DWHP.

### 4.4. Use and Opportunity to Use

The possibility of using on-site offers is given for 70.3%, and slightly higher for digital offerings at 74.9%. For the latter, complete agreement is more likely to be expressed by people over 45 years of age (Chi2 test, *p* = 0.011).

**Figure 4:**
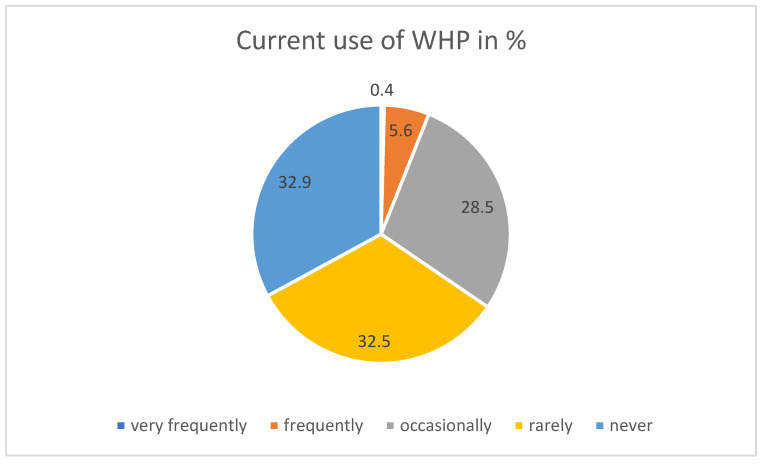
Current use of WHP (on-site offer).

A total of 67.1% of participants used WHP offers from very frequently to rarely, with a tendency toward people without patient contact (Chi2 test, *p* = 0.000). Higher use was reported by 34.5% who used WHP offers between very frequently and occasionally (see Figure 4), frequently in particular by people with core working hours (Chi2 test, *p* = 0.020). It was rarely or never used by around 32% of respondents (32.5% and 32.9%, respectively), and significantly rather never by people working day and night shifts (same Chi2 test). Regarding future plans, 90% stated that they wanted to use WHP.

**Figure 5:**
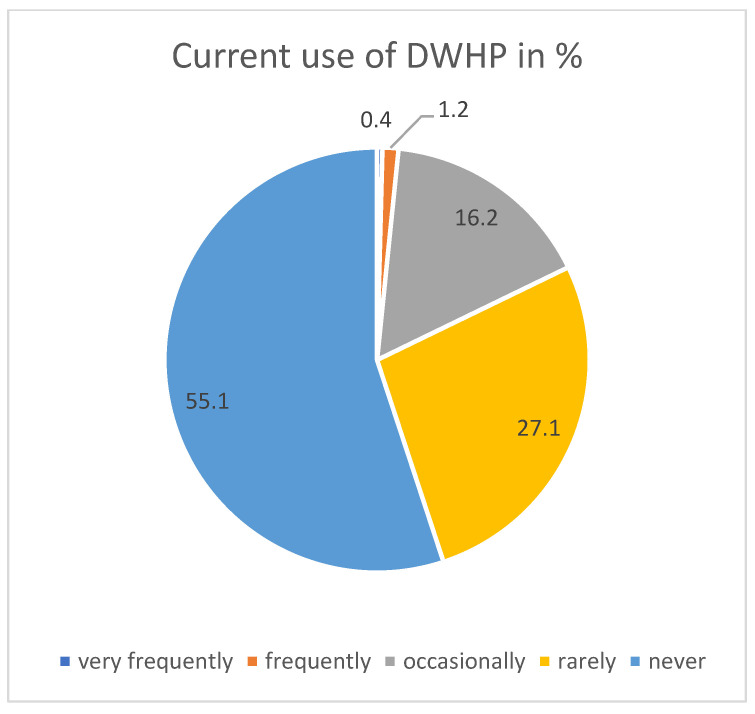
Current use of DWHP.

The usage data on DWHP are currently lower compared to on-site WHP. A total of 44.9% respondents used the offer (including rarely), here too tending to be people without patient contact (Chi2 test, *p* = 0.002). Between very frequently and occasionally (including three response levels), 17.8% used DWHP (see Figure 5). Therefore, H1a—DWHP use is high—is not confirmed.

**Figure 6:**
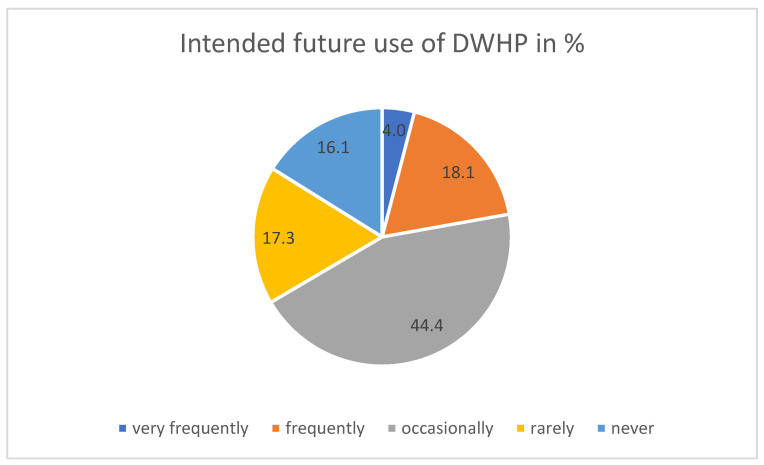
Intended future use of DWHP.

However, the reported rate of future use is rather high: 83.9% planned to use it, confirming H1b—Intended use of DWHP is high: 83.9% stated the intention to use DWHP in the future, with 4% very frequently, 18.1% frequently, 44.4% occasionally and 17.3% seldom (see Figure 6).

Future plans regarding DWHP use are highly connected to working time regimes and current organizational aspects measured in the evaluation of psychosocial burden at the workplace. Those who reported that there was not enough personnel in their work environment (Chi2 test, *p* = 0.022) and those who could rarely decide when to take a break (Chi2 test, *p* = 0.037) were likelier to plan for more DWHP use. Also, those who stated that there was not enough time for all tasks were significantly more likely to plan to use DWHP very frequently in the future or to not intend to use it at all (Chi2 test, *p* = 0.003).

### 4.5. DWHP: Opportunities and Limitations

Below, the opportunities and limitations of DWHP according to the participants are described.

DWHP services were practical for 67.4% [53.1%] of respondents because they can be used flexibly regarding location. Time-related flexibility was mentioned by 65.7% [53.5%], and according to the Chi2 test this mainly applies to those without patient contact (*p* = 0.009). A total of 66.4% said that using DWHP saved time. The Chi2 test shows that this tends to be less important for people without patient contact (*p* = 0.038). Moreover, 55% rated DWHP as easier to use than WHP.

In total, this confirms H2 as the perceived benefits are higher in the second survey compared to the first.

In the evaluation survey, 21.1% [19.9%] of respondents stated that they could not participate in DWHP due to their technical equipment. In addition, 15.1% [12.9%] could not participate due to their technical skills. This was stated mainly by people who were older than 45 years (Chi2 text, *p* = 0.000). The working spaces were an obstacle for 39.5% [38.6%].

Taken together, this indicates that H3—The perceived barriers for DWHP use decrease over time—is not confirmed.

Regarding the limits of DWHP use, men tended to be more optimistic and report fewer problems (Chi2 test, *p* = 0.044 for working spaces, 0.014 for technical skills, 0.027 for technical equipment). The deviations in the values compared to 2022 are small, but nevertheless noticeable. There appears to be a need for training and support, especially for women.

### 4.6. Further Wishes

Regarding interests, all of the provided options received high ratings (general improvement in working conditions, stress management, sports/physical exercise, nutrition, lectures and information events, health day, group activities, spiritual offers).

Open suggestions for improvement can be clustered in timing aspects, the offer design, specific spatial concerns and related information. Below, only those that are applicable to a broad range of organizations are described.

- Timing aspects: The announcement of offers should start as early as possible (approx. 6 months in advance) and all working hours covered (including evenings). Short programs (15 min weekly) are welcomed.
- Offer design: offers should be repeated, and either provided online or at all locations of the hospital; physical activity offers should cover all fitness levels.
- Information: supervisors and managers should be required to communicate the (D)WHP offer and motivate employees to participate.

## 5. Discussion

This study focused on describing the implementation of DWHP in a hospital in Austria which is already actively engaged in WHP. Digitalization was seen as a general necessity in the HR context, but also to decrease inequalities of access possibilities to WHP. A highly target-group-oriented approach was chosen to ensure the new offer was aligned to the employees’ needs. However, it is important to keep in mind that the setting must be conducive for health, as well [36]. While reporting on the baseline data and the evaluation, the focus was on the latter to test five hypotheses, of which three were confirmed.

DWHP is rated favorably by the employees (H1: confirmed), but they are very much more in favor of on-site offers, underlining that digital interventions should only constitute an addition to regular WHP initiatives. This tendency is partly reflected in the currently lower rate of DWHP use (H1a: high use of DWHP is not confirmed) compared to actively engaging in WHP. However, the intended use is pronounced (H1b: high intended future use is confirmed), especially for those who experience high time-related pressure in their daily work. This confirms the initial reasoning for introducing DWHP in the organization.

This study shows that the benefits of DWHP were stated by more participants in the second survey, confirming H2. However, barriers to using DWHP did not decrease, which is why H3 was not confirmed. This could be due to a higher awareness of potential barriers, but also highlights the training and assistance needs of employees. Working on barrier reduction should be a major focus to ensure that the benefits of DWHP can be reaped. While the very positive ratings show that there is a significant amount of potential for salutogenetic transformation, the connections to time pressure also require improvements in the general working conditions. Though many of the project targets have been reached, staff with rigid working times and those with direct patient interaction need to be focused on even more. Overall, flexibility (in terms of time and location) appears to be more relevant than saving time. Interestingly, many benefits of DWHP were more relevant for those without patient contact. As these are traditionally seen as those who can better plan for WHP participation, this needs to be further investigated.

The results show that the demand for DWHP is high and likely to rise further. As Bleier and colleagues [85] highlight, the demand especially for psychological health promotion is high and the offer is likely to be expanded. However, they also raise the issue of high dropout rates in DWHP offers, which may be due to a vicious cycle of lack of time and exhaustion. This may call for increasing precision prevention [108], e.g., with apps [36]. Recent research indicates that DWHP is not only effective for health promotion and prevention [39]. The use of health apps at the workplace can even improve retention rates [43]. As all health issues can be addressed [30], it may be advisable to investigate the current state of burdens and resources and to then focus on the major topics. Then, the impact may be even greater. However, internal project and organizational leadership has to be very committed [109], which is the case for this project. Moreover, data security has to be ensured.

Sallinen [24] highlights the importance of stakeholder trust in the context of DWHP. As indicated above, placing health concerns in the hands of the employer (digitally) may involve data security issues [41,110]. By including third-party applications into the range of options, privacy aspects may be easier to accommodate as the employer is not the one who offers the measure [111]. One way to do so is by creating a pool of DWHP initiatives and sharing them via networks of organizations. When recorded and posted on the intranet or another site, the offers could even be offered for a longer time, which creates a second benefit to cost sharing in a network [112].

Workplace health initiatives hold the danger of reducing the individual perception of responsibility for health, leading to less autonomy. Even if autonomy is kept, and for example used in the form of not participating in (D)WHP, falling ill may lead to being held even more accountable [113]. This could lead to people opting in even though they would rather not, which again decreases autonomy. The same holds true for information and communication technology and its regular use just as DWHP. While ICT allows for remote work, increasing temporal and spatial autonomy, it can be tied to the expectation of availability, limiting behavioral options and contradicting autonomy [114,115]. Temporal and spatial autonomy, however, are needed as slack and distancing to make innovation and recovery possible. Therefore, DWHP should also not lead to negative work–home boundary spilling or add pressure.

### 5.1. Future Research Requirements

Blind spots in WHP research and consequently DWHP are unintended consequences as, for example, creating stress due to WHP participation [57] or reducing additional payments due to demanding working contexts (heat, dust, noise, …) when conditions are improved. Furthermore, unexpected associations like the physical activity paradox must be considered, stating that occupational physical activity is related to higher mortality. This is because higher degrees of physical activity at work are related to lower job qualification levels [116]. Physical activity interventions in (D)WHP would have to take this into account, differentiating between what is required by whom. As Tengland [117] highlights, the type of job needs to be considered, especially in the context of workability, as some lines of work require higher degrees of certain aspects of health, together with relevant competences. A recent systematic review on DWHP underlined that employees’ health literacy is crucial for achieving positive effects [39]. This implicitly calls for targeted workplace health promotion. Moreover, a more detailed analysis of the benefits of DWHP is required, including usability issues (e.g., understandability, applicability, integration into the daily routine), barriers to use DWHP compared to WHP, and employee-perceived health impacts. In this context, the effects of engaging in specific types of DWHP require a detailed analysis. Higher-order statistical analyses like structural equation models could be used to investigate interrelationships of barriers, offers, and impacts. Also, potential unintended effects (e.g., reduction in interaction with colleagues compared to on-site offers) and how to avoid these need to be investigated.

“[T]he question arises as to the chances of realizing an opportunity- and health-oriented design of work. These are characterized by the tension between ethical orientation and economic benefit considerations (…)” [118] (p. 9). Since digital offers are typically much cheaper, this opens new opportunities that organizations should consider. In line with this, future research should evaluate the employer’s perspective on DWHP and analyze reduced costs and impacts on organizational performance data. As the situation during the pandemic highlighted, individual career choices and the staffing and thus existence of entire economic sectors are affected by the degree to which organizations can ensure employee health in a broad sense [119]. Improving the health and safety climate by general setting-oriented initiatives and targeted offers is promising.

### 5.2. Limitations

This study focused on providing an overview of the demographic differences regarding DWHP perception and does not come without limitations. First, we considered one organization only, and in a very specific sector (healthcare). Moreover, we only investigated employees and no other stakeholders. Nevertheless, this provides the opportunity to survey a population with very diverse educational backgrounds, jobs and working times. Thus, the results are of interest not only for other hospitals, but also large organizations in which employees have very different job types and schedules. However, we highly advise for studies in other sectors, as well, and in different nations. Moreover, we advocate for studies that differentiate in more detail regarding demographic criteria, which was not possible in this case due to ethical concerns, e.g., regarding distinguishing in more detail between age groups. Also, identical scaling should be ensured in case several surveys are used.

Second, though we can report on two investigations, we cannot match the answers of the surveys as anonymity concerns did not allow for using personal codes. However, this approach can reduce potential biases like social desirability. Future studies may consider the option of personal codes and provide data on within-person changes using various DWHP offers. Also, group comparisons of WHP and DWHP offers on identical topics and the outcomes regarding statistical twins may be highly interesting. This may also offer the possibility to reduce potential nonresponse bias and people could be matched based on their digital competences. Currently, those interested in WHP and DWHP may have participated in higher numbers, so an analysis of non-respondents may be a fruitful avenue for research.

Third, we employed quantitative surveys with only a few open questions. Though the response rate is rather high compared to the sample size, it is very likely that there are patterns that require in-depth interaction with the target group. Therefore, we encourage studies adding qualitative data stemming from interviews or observing employees using DWHP.

Fourth, we did not ask about employment history and whether employees had already worked for an employer with a DWHP offer, as this is a very recent development. However, future studies should take this into account.

## 6. Conclusions

Many organizations offer workplace health promotion (WHP) [73,120], and increasing stress and strain at the workplace suggests this is much needed. As can be seen from the results of this case study, DWHP does have the potential to contribute to the sustainable development and transformation of organizations via the high interest it generates. Most of the respondents intended to use DWHP in the future, which provides a unique opportunity for organizations to improve the health and wellbeing of their employees via digital health promotion offers. Designing attractive jobs will be crucial for the further development of many industries, as current personnel shortages highlight. Changes regarding how organizations treat employees can thus be expected and are hypothesized to result in advantages for all. Whether this is mainly altruistically or financially motivated, or both, is an open ethical question. Its perceived answer can influence the reaction of the stakeholders due to the importance of credibility of corporate actions regarding health promotion. The latter is improved by providing opportunities for the true co-creation of health programs at the workplace [23,57,121], and evaluated against the ideas held of what organizations should look and be like, provide and stand for. Thus, compliance with laws and societally expected working standards, co-creation, participation and target group orientation must range among the principles for creating a healthy job. Nevertheless, the role of organizations in health needs to be discussed.

This study is about a project on digital health promotion for employees in a hospital in Austria, thus referring to sustainable HR aided by digitalization. It is important to note here that employees may have a different impression of the WHP offer than employers [16]. Individual capabilities must be considered just as much as motivational issues, together with ensuring the opportunity to participate [79], which is much improved by digital offers in general and even further profits from investigating the employees’ wishes and needs. The creation of salutogenic structures and processes in organizations requires an intensive dialogue with internal and external stakeholders, just as the choice of (digital) interventions does [38]. In accordance with comprehensive descriptive management models, the topics to be considered include values, concerns and resources [38,56,122]. This study can serve as a first basis for organizations interested in supplementing WHP with digital aspects.

## Figures and Tables

**Table 1:** Demographics in surveys 1 and 2.

Item	2022	2023
Gender	Not surveyed	85.2%: female 14.8%: male 0%: diverse
Age	≤30: 21.2% 31–45: 36.9% 46–55: 19.5% >56: 7.9% Missing values: 14.5%	≤45: 55.5% >45: 44.1% Missing values: 0.4%
Occupational group	Nursing: 50.2% Medical service professions: 9.1% Administration: 16.2% Medical professions: 7.1% Services/technical occupations: 2.5% Missing values: 14.9%	Nursing: 55% Medical service professions: 18.2% Administration: 16.1% Medical professions: 7.9% Services/technical occupations: 2.9%
Patient contact	Not surveyed	Yes: 87.8% No: 12.2%
Type of patients cared for	Not surveyed	Acute: 27.4% Chronic: 16.9% Both: 55.7%
Extent of employment	≤20 h/week: 9.1% >20 h/week: 76.3% Missing values: 14.5%	≤20 h/week: 11.3% >20 h/week: 88.7%
Schedule	Shift work: 10.4% Only day duties: 45.2% Day and night duties: 28.6% Missing values: 15.8%	Shift work: 17.4% Only day duties: 44.9% Day and night duties: 27.1% Core working hours: 10.7%
On-call duties	Not surveyed	Yes: 10.6% No: 89.0% Missing values: 0.4%
Education	Statutory education: 2.5% Vocational training: 33.6% A levels: 22.8% University/university of applied sciences: 25.3% Missing values: 15.8%	Not surveyed
Length of employment	Not surveyed	≤3 years: 36.9% >3 years: 63.1%

## Data Availability

Restrictions apply to the dataset due to an ongoing study.

## Appendix A. Questionnaire of First Survey

Note: The survey was conducted in German. The items were translated by the first author.

**PART ONE: General questions in the work context**

1. **Digitalization in the organization** (agree/rather agree/neither-nor/rather disagree/disagree)
  - Regarding digitalization, the organization is on the pulse of time.
  - I know the homepage of the organization.
  - I like the homepage of the organization.
2. **Digitalization in the own working environment** (agree/rather agree/neither-nor/rather disagree/disagree)
  - I’m personally interested in digitalization.
  - I’m confronted with digitalization in my daily work.
  - At work, I have access to digital devices like PC, laptop, etc.
  - I work with digital devices that are personally assigned to me.
  - In the foreseeable future, my daily work will be (even) more digitalized.
  - My daily work requires me to use information and communication technology (e-mails, text messages, software, etc.)
  - My skills for using information and communication technology are sufficient.
  - The instructions for using information and communication technology in my everyday professional life are helpful.
  - The use of information and communication technology makes my everyday professional life more efficient.
  - The use of information and communication in my everyday professional life
    ○ Improves work quality;
    ○ Improves patient safety;
    ○ Works smoothly;
    ○ Creates additional work.
  - I find the extent of information and communication technology in my everyday professional life wearing.
  - I would like to receive training for the use of information and communication technology.
    ○ I have the following suggestions for trainings: open response.
3. **Wishes** (agree/rather agree/neither-nor/rather disagree/disagree)
  - I already get along well with the new employee portal.
  - I would like to receive training for the use of the new employee portal.
  - I would like to have e-learning options via the new employee portal.
    ○ Specific ideas for contents: open response.
  - I would like to have additional content in the new employee portal: open response.

**PART TWO: WHP and DWHP**

1. **General questions regarding WHP and digitalization** (agree/rather agree/neither-nor/rather disagree/disagree)
  - I know where to find information on the WHP offer in the employee portal.
  - I use the employee portal to get information on WHP.
  - The digital information options on WHP are easy to use.
  - The digital registration for WHP offers are easy to use.*
  - Digital WHP offers are a good addition to non-digital offers.*
  - In my opinion, WHP should only take place “live”.
  - In my opinion, only these specific WHP offers should be digitalized: open response.
2. **Use of DWHP** (agree/rather agree/neither-nor/rather disagree/disagree)
  - I would use digital live offers focused on exercise (e.g., shared exercise breaks done as online meeting).
  - I would participate in digital live lectures when I’m interested in the topic and do have time.
  - I would watch recordings of occupational health-related information (e.g., ergonomics for the home office).
  - I would use digital live consultation of occupational medical personnel (e.g., ergonomics for the home office).
  - I would only use live offers.
  - I would only use recordings.
  - I would use both live offers and recordings.
3. **Barriers for DWHP use** (agree/rather agree/neither-nor/rather disagree/disagree)
  - I have so many digital parts in my daily work that I do not want digital WHP offers. *
  - Participating in digital WHP offers would not be possible for me
    ○ Due to my technical equipment *;
    ○ Due to my technical skills;
    ○ Due to the working space *.
4. **Benefits of DWHP use** (agree/rather agree/neither-nor/rather disagree/disagree)
  - Digital WHP offers would be practical because I could use them flexibly regarding location.
  - Digital WHP offers would be practical because I could use them flexibly regarding time (travelling times, waiting, …). *
5. **Attractiveness of digital offers** (very attractive/rather attractive/neither-nor/rather not attractive, not attractive)
  - How attractive would digital LIVE-offers of WHP be for you in the following areas?
    ○ Dietary habits;
    ○ Exercise;
    ○ Ergonomics;
    ○ Mental health;
    ○ Stress management;
    ○ Mindfulness;
    ○ Resilience;
    ○ Non-violent communication;
    ○ Workplace design (at the organization);
    ○ Optimizing movement sequences in daily work;
    ○ Workplace design (at the home office);
    ○ Other: open response.
  - How attractive would recordings of digital offers of WHP be for you in the following areas?
    ○ Dietary habits;
    ○ Exercise;
    ○ Ergonomics;
    ○ Mental health;
    ○ Stress management;
    ○ Mindfulness;
    ○ Resilience;
    ○ Non-violent communication;
    ○ Workplace design (at the organization);
    ○ Optimizing movement sequences in daily work;
    ○ Workplace design (at the home office);
    ○ Other: open response.
  - I would like to receive training for using digital offers of WHP.

**PART THREE: Demographic questions**

- Age group: ≤30, 31–45, 46–55, >56.
- Occupational group: nursing personnel, medical personnel, medical service professions, administration, technical/service personnel.
- Location: locations of the organization.
- Extent of employment: ≤20 h/week, >20 h/week.
- Schedule: only day duties, day and night duties, shift work.
- Education: statutory education, vocational training, A levels, university/university of applied sciences.

* see [14]

## Appendix B. Questionnaire of Second Survey

Note 1: The survey was conducted in German. The items were translated by the first author.

Note 2: Italics indicate items that were also used in the first survey. In case these were adapted to evaluate the offer, this is highlighted in bold.

**PART ONE: WHP and DWHP** (applies/rather applies/rather not applies/does not apply)

- Workplace Health Promotion (WHP) is a generally important topic.
- I find WHP important for me personally.
- I know where to find information on the WHP offer.
- Information on the WHP offer reaches everyone in the organization.
- I see general improvements in working conditions in the organization due to WHP.
- Using WHP offers on-site is possible for everyone.
- Using digital WHP offers is possible for everyone.
- I know which digital WHP offers are available in the organization.
- *I know where to find information on the WHP offer in the employee portal.*
- *I use the employee portal to get information on WHP.*
- *The digital information options on WHP are easy to use.*
- *The digital registration for WHP offers are easy to use.**
- *Digital WHP offers are a good addition to non-digital offers.**
- *In my opinion, WHP should only take place “live”.*
- I feel well prepared for digitalization in my work context.
- *Digital WHP offers **are** practical for me because I **can** use them flexibly regarding location.*
- *Digital WHP offers **are** practical for me because I **can save** time (travelling times, waiting, …).**
- For me, digital WHP offers are easier to use than those on-site.
- *Digital WHP offers **are** practical because I **can** use them flexibly regarding time (travelling times, waiting, …).*
- *I have so many digital parts in my daily work that I do not want digital WHP offers.**
- *Participating in digital WHP offers **is** not possible for me*
  ○ Due to my technical equipment*;
  ○ Due to my technical skills;
  ○ Due to the working space.*

**PART TWO: Use of WHP and DWHP** (very frequently/frequently/occasionally/rarely/never)

- How frequently do you currently use WHP offers on-site?
- How frequently do you currently use digital WHP offers?
- How often would you like to use WHP offers on-site in the future?
- How often would you like to use digital WHP offers in the future?

**Part THREE: Interest in specific offers** (very interesting/rather interesting/neither-nor/rather not interesting/not interesting at all)

- How interesting are the following areas of WHP for you personally?
- Sports and Exercise.
- Nutrition.
- Stress management.
- General improvements of working conditions.
- Lectures and Information events.
- Group activities.
- Spiritual offer.
- Health day.
- Other: open response.

**PART FOUR: Rating and Satisfaction** (very satisfied/rather satisfied/rather unsatisfied/very unsatisfied)

- How satisfied are you with the WHP offer at the organization in general?
- How satisfied are you with the WHP offer on-site of the organization?
- How satisfied are you with the digital WHP offer of the organization?
- Which suggestions do you have for improving the WHP offer at the organization? Open response.

(various scales to evaluate psycho-social burden at the workplace, not reported here)

**LAST PART: Demographic questions**

- Patient contact: yes/no.
- Type of patient cared for: acute, chronic.
- Schedule: shift work, only day duties, day and night duties, core working hours.
- On-call duty: yes/no.
- Occupational group: medical personnel, nursing personnel, administration, medical service professions, technical/service personnel.
- Extent of employment: ≤20 h/week, >20 h/week.
- Length of employment at the organization: ≤3 years, >3 years.
- Age group: ≤45, >45.
- Gender: male, female, diverse.

* see [14]
